# A Novel Location Source Optimization Algorithm for Low Anchor Node Density Wireless Sensor Networks

**DOI:** 10.3390/s21051890

**Published:** 2021-03-08

**Authors:** Zhongliang Deng, Shihao Tang, Xiwen Deng, Lu Yin, Jingrong Liu

**Affiliations:** School of Electronic Engineering, Beijing University of Posts and Telecommunications, Beijing 100876, China; dengzhl@bupt.edu.cn (Z.D.); dengxiwen@bupt.edu.cn (X.D.); inlu_mail@bupt.edu.cn (L.Y.); jingrongliu@bupt.edu.cn (J.L.)

**Keywords:** cooperative localization, location source optimization, fuzzy comprehensive evaluation, DCPCRLB

## Abstract

Location information is one of the basic elements of the Internet of Things (IoT), which is also an important research direction in the application of wireless sensor networks (WSNs). Aiming at addressing the TOA positioning problem in the low anchor node density deployment environment, the traditional cooperative localization method will reduce the positioning accuracy due to excessive redundant information. In this regard, this paper proposes a location source optimization algorithm based on fuzzy comprehensive evaluation. First, each node calculates its own time-position distribute conditional posterior Cramer-Rao lower bound (DCPCRLB) and transfers it to neighbor nodes. Then collect the DCPCRLB, distance measurement, azimuth angle and other information from neighboring nodes to form a fuzzy evaluation factor set and determine the final preferred location source after fuzzy change. The simulation results show that the method proposed in this paper has better positioning accuracy about 33.9% with the compared method in low anchor node density scenarios when the computational complexity is comparable.

## 1. Introduction

The Internet of Things (IoT) is a booming new industry and wireless sensor networks (WSN), as the perception layer of the IoT system [1], provide data collection [2], information transmission [3], scene recognition [4] and other functions to ensure the normal operation of the networked system. Wireless sensor networks can replace humans working in harsh natural environments and complete complex and tedious tasks. They are widely used in precision agriculture [5], elderly care [6], air monitoring [7], smart home [6], disaster supervision [8] and many other fields.

Taking into account the actual needs of IoT systems, WSN usually have the characteristics of real-time communication [9], random and irregular distribution of nodes [10], dynamic topology [11], large scale [12], complex deployment environment [13], etc., which brings a certain degree of difficulty to information collection, processing and analysis. Obtaining accurate location and time information of data sources is a prerequisite for sensor network analysis and application data [9,10,11,12,13]. Therefore, the positioning and time synchronization technology of WSN is an important part of the application of the IoT, which has attracted the enthusiasm of researchers at home and abroad.

**Figure 1:**
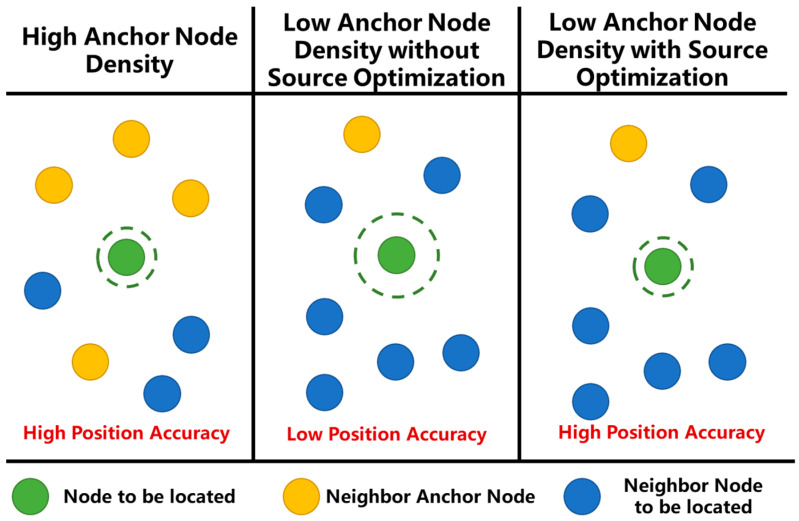
Different position accuracy with different anchor node density. Under low anchor node density, the number of neighbor nodes to be located in the position source increases, and the uncertainty of the localization information of the position source increases, resulting in a decrease in the position accuracy of the node to be located.

Common positioning principles include Approximate Perfect Point-In-Triangulation (APIT) [14], distance vector hop (DV-Hop) [15], received signal strength indicator (RSSI) fingerprint [16] that are range-free method, and RSSI ranging [17], times of arrival (TOA) [18] and time difference of arrival (TDOA) [19] based on ranging information. The positioning accuracy of the range-base localization algorithms are usually better than the range-free positioning localization algorithms. The localization algorithms based on TOA ranging information is one of the main research directions of sensor network positioning due to its low cost and high positioning accuracy [20]. However, due to the relatively high cost of anchor nodes, it is difficult to deploy them in large numbers in actual applications. Cooperative localization method can rely on the location information and communication channels of other nodes to provide coordinated information to improve the positioning performance of the system [21]. However, the coordinated information in the scene of low anchor node density may also cause a decrease in positioning accuracy [22], as shown in Figure 1.

Therefore, it is necessary to optimize and screen out the location source that is helpful to improve the positioning performance. The main contributions of this paper are summarized as follows:

(1) Frist of all, the system model of low anchor node density is defined. Nodes calculate location and time skew with TOA method in this model.
(2) Then, a novel location source optimization algorithm is proposed for low anchor node density scenario. In the proposed method, a location source select structure is established with fuzzy comprehensive evaluation. Distribute conditional posterior Cramer-Rao lower bound (DCPCRLB), distance measurement and direction angle is considered as the most significant factors to select location source.
(3) The validity and rationality of the proposed method are verified by experiments.

The structure of this paper is as follows: Section 1 is the introduction, which summarizes the background knowledge and explains the significance of studying the location source select in cooperative localization. Section 2 lists the related work of this research direction in recent years. Section 3 gives out the system model of cooperative localization in low anchor node density, then proposes a novel location source select method based on fuzzy comprehensive evaluation. Section 4 shows the simulation scenario, analyses the performances of the proposed method in this paper and discusses the future work of this studying. Section 5 presents a summary of the research content of this paper.

## 2. Related Work

In recent years, anchor node selection has been a research topic in cooperative localization. Researchers pay lot of attention to anchor node selection, but the ambiguity of nodes position and distance measurement leads to a low accuracy of node position. So far, domestic and foreign experts and scholars have done a lot of research on anchor node selection from different aspects and have achieved good results. The main work is as follow:

In [23], a localization method called mobile-beacon based iterative localization (MBIL) is proposed. In this method, the position confidence of the node is calculated with the number of iterations, the residual energy and the deviation degree error of the localized node’s estimated location. The confidence is used to optimize the location source, and could achieve a high positioning performance is achieved in a short time. 

Reference [24] analyzes the localization error caused by the selection of anchor nodes first. Based on above analysis conclusion, they proposes an improved least square localization algorithm based on the selection of anchor nodes with distance clustering (LSL-DC). With distance clustering, the anchor nodes are chosen. The simulation results indicate LSL-DC algorithm can improve the localization precision.

In [25], distance measure errors are also considered as the most significant factor which affect position accuracy, a optimizing method called node segmentation with improved particle swarm optimization (NS-IPSO) is proposed to filter the positioning source by the distance between nodes and the communication frequency, so as to avoid large distance measurement errors and improve positioning accuracy.

Reference [26] considers a localization problem in non-uniformly and holes in application environment, which affect the accuracy of distance estimation and causes large position errors in node positioning. They proposed a localization method called boundary-based anchor selection method for WSNs node localization (BASL). In this method, nodes first explore WSN connectivity to confirm whether they are boundary region nodes. Then, the node to be locate selects anchor nodes by checking the number of boundary region nodes in their shortest path between itself and anchor nodes. The results show that the BASL method can alleviate localization error which is caused by the hole in the scenarios.

Reference [27] analyzed the impact of horizontal dilution of precision (HDOP) on positioning accuracy in underwater scenes, and selected the node with the smallest HDOP as the location source to obtain the highest positioning accuracy, which called generalized second-order time-difference-of-arrival (GSTDOA) algorithm. 

In [28], an enhanced three-dimensional DV-hop algorithm is proposed, which enhance its location accuracy. Coplanarity is used to select an optimal set of beacon nodes around an unidentified node for its location estimation.

Reference [29] proposed social network analysis based localization technique with closeness centrality (SNA-CC). Closeness centrality is obtained by calculating the average distance value between the node and all its neighbor nodes. This paper uses this as the importance evaluation criterion of nodes to screen the nodes. After screening, the positioning accuracy has been improved to a certain extent, and its essence is still in the screening of distance measurement errors.

In [30], a method called dynamic reference selection-based self-localization algorithm (DRSL) is proposed which combine location sources with the smallest least square error were selected to achieve the best positioning accuracy.

The following Table 1 summarizes the above-mentioned location source selection algorithm literature, the screening factors used, and the method of fusion between factors, and compares them with the method proposed in this article.

The above methods only consider part of the factors that affect the positioning performance, and do not include all factors in the positioning source optimization framework. The main contribution of this paper is a novel optimization algorithm of the fuzzy comprehensive evaluation [31] framework, which forms a flexible and fast positioning source selection framework. At the same time, it is based on many factors to select the location source such as the DCPCRLB considering the influence of clock, distance observation, direction angle, etc.

## 3. Location Source Selection Algorithm Based on Fuzzy Comprehensive Evaluation

In this section, first a two-dimensional positioning scene is shown, and the positioning principle and positioning method used in this article are basically explained. Then the DCPCRLB is introduced to pave the way for the proposed location source optimization method. Finally, the node selection algorithm proposed in this article is introduced, and the steps and operation process of the algorithm are introduced.

**Figure 2:**
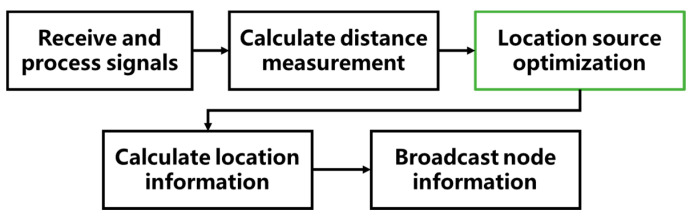
Flow chart of cooperative localization with location source optimization.

As shown in the Figure 2, the positioning node selection is an optional step after calculating the distance measurement in the overall positioning process and before the positioning solution. The addition of positioning node selection can reduce the computational complexity of subsequent steps, thereby reducing the time-consuming of the overall positioning process. At the same time, since the node selection screens low-quality positioning sources, the positioning accuracy can be improved.

### 3.1. System Model

In the actual layout, the anchor nodes in the same scene are usually at the same height, so there is a big error in height measurement. Height measurement is usually achieved by other methods, so two-dimensional scenario is considered. The positioning scene includes anchor nodes and nodes to be positioned. The position vector of node i at time l is represented by $x_{i,l}={\left[\begin{array}{cc} x_{i,l} & y_{i,l} \end{array}\right]}^{T}$, and the clock slope is represented by $a_{i,l}≜\left({\tilde{t}}_{i,l}-{\tilde{t}}_{i,l-1}\right)/\left(T_{l}-T_{l-1}\right)$, ${\tilde{t}}_{i,l}$ represents the local time of node i, and $T_{l}$ represents the real time. Assume that the anchor node time is synchronized with the real time, that is, for anchor node i, $a_{i,l}=1$. The estimated vector of the node i to be located at time l is $\theta_{i,l}={\left[\begin{array}{ccc} x_{i,l} & y_{i,l} & a_{i,l} \end{array}\right]}^{T}$, its line-of-sight neighbor node set is defined as $N_{i,l}$. The set of vectors to be estimated for all nodes to be located at time l is $\Theta_{l}=\left\{\cdots ,\theta_{i,l},\cdots \right\}$.

Node i obtains TOA observations by receiving information from neighboring node j at time l as follows:

(1) $${\tilde{r}}_{ij,l}=ǁx_{i,l}-x_{j,l}ǁ+cT\left(a_{i,l}-a_{j,l}\right)+n_{ij,l},$$

where $ǁx_{i,l}-x_{j,l}ǁ$ represents the Euclidean distance between node i and node j at time l, c is the speed of light, T is the time difference between adjacent moments, and $n_{ij,l}$ is TOA observation noise, assuming that it conforms to Gaussian distribution as $n_{ij,n}\sim N\left(0,\sigma_{r}^{2}\right)$. Then the probability density function of ${\tilde{r}}_{ij,l}$ with respect to $\theta_{i,l},\theta_{j,l}$ can be expressed as:

(2) $$p\left({\tilde{r}}_{ij,l}|\theta_{i,l},\theta_{j,l}\right)=\frac{1}{\sqrt{2\pi \sigma_{r}^{2}}}\exp\left(\frac{{\left({\tilde{r}}_{ij,l}-ǁx_{i,l}-x_{j,l}ǁ-cT\left(a_{i,l}-a_{j,l}\right)\right)}^{2}}{2\sigma_{r}^{2}}\right),$$

Assuming that the change process of the vector $\theta_{i,l}$ to be estimated at node i conforms to the Gaussian Markov process [32], according to the Bayesian formula:

(3) $$p\left(\theta_{i,l}|{\tilde{r}}_{ij,l}\right)\propto p\left(\theta_{i,l}|\theta_{i,l-1}\right)\prod_{j\in N_{i,l}}p\left({\tilde{r}}_{ij,l}|\theta_{i,l},\theta_{j,l}\right),$$

According to the MAP criterion, the estimated value ${\hat{\theta }}_{i,l}$ of the vector $\theta_{i,l}$ to be estimated at node i can be expressed as:

(4) $$\begin{array}{cc} {\hat{\theta }}_{i,l} & =arg\underset{\theta_{i,l}}{\max}p(\theta_{i,l}|{\tilde{r}}_{ij,l},\theta_{j,l})\\ & =arg\underset{\theta_{i,l}}{\max}p\left(\theta_{i,l}|\theta_{i,l-1}\right)\prod_{j\in N_{i,l}}p\left({\tilde{r}}_{ij,l}|\theta_{i,l},\theta_{j,l}\right), \end{array}$$

### 3.2. Distributed Cramer-Rao Lower Bound

Cramer-Rao lower bound (CRLB) is the inverse matrix of the Fisher Information Matrix (FIM) of the random variable $\theta_{i,l}$, and is a theoretical lower bound of the mean square error of the target state estimation. The conditional posterior CRLB (CPCRLB) of the estimated state ${\hat{\theta }}_{i,l}$ of node i can be expressed as:

(5) $$\begin{array}{cc} MSE\left({\hat{\theta }}_{i,l}|{\tilde{r}}_{ij,l}\right) & =E\left\{\left[{\hat{\theta }}_{i,l}-\theta_{i,l}\right]{\left[{\hat{\theta }}_{i,l}-\theta_{i,l}\right]}^{T}|{\tilde{r}}_{i,l}\right\}\\ & \geq F^{-1}\left({\hat{\theta }}_{i,l}|{\tilde{r}}_{i,l}\right), \end{array}$$

Among them, $F\left({\hat{\theta }}_{i,l}|{\tilde{r}}_{i,l}\right)$ represents the conditional FIM of the target state estimated value ${\hat{\theta }}_{i,l}$. [33] gives the global Fisher information matrix F(l) iterative calculation formula of centralized CPCRLB, which can be applied to a centralized network structure with a central fusion center to realize a centralized cooperative localization. This article is aimed at a distributed network structure, and each node cannot obtain the global Fisher information matrix. Therefore, we adjust the above formula to obtain the local Fisher information matrix iterative formula corresponding to the DCPCRLB:

(6) $$F_{i}\left(l\right)\approx B_{i}^{22}\left(l-1\right)-B_{i}^{21}\left(l-1\right){\left(B_{i}^{11}\left(l-1\right)+F_{i}\left(l-1\right)\right)}^{-1}B_{i}^{12}\left(l-1\right),$$

where:

(7) $$B_{i}^{11}\left(l-1\right)=E\left(-{∆}_{\theta_{i,l-1}}^{\theta_{i,l-1}}\ln p\left(\theta_{i,l}|\theta_{i,l-1}\right)\right),$$

(8) $$B_{i}^{12}\left(l-1\right)=E\left(-{∆}_{\theta_{i,l-1}}^{\theta_{i,l}}\ln p\left(\theta_{i,l}|\theta_{i,l-1}\right)\right)={\left(B_{i}^{21}\left(l-1\right)\right)}^{T},$$

(9) $$B_{i}^{22}\left(l-1\right)=E\left(-\Delta_{\theta_{i,l}}^{\theta_{i,l}}\left(\ln p\left(\theta_{i,l}|\theta_{i,l-1}\right)+\ln p\left({\tilde{r}}_{i,l}|\theta_{i,l}\right)\right)\right),$$

where ${∆}_{\Theta_{l-1}}^{\Theta_{l}}=\nabla_{\Theta_{l-1}}\nabla_{\Theta_{l}}^{T},\nabla_{\Theta_{l}}={\left[\cdots ,\frac{\partial }{\partial \theta_{i,l}},\cdots \right]}^{T}$. F(l) is a global Fisher information matrix. The DCPCRLB of node i can be obtained by inverting the Fisher information matrix, that is, $CRLB\left(\theta_{j,l}\right)=F_{i}^{-1}\left(l\right)$.

### 3.3. Location Source Optimization Algorithm

In a 2-dimensional wireless sensor network, the node to be located needs to establish communication with at least three anchor nodes to complete positioning. In the location-time joint estimation problem, it is necessary to establish communication with at least four anchor nodes to complete the calculation of the vector to be estimated. In the scenario of low anchor node density, the cooperative localization method provides neighbor nodes as pseudo-anchor nodes for the node to be located, but the pseudo-anchor node itself has low accuracy as the node to be located, and the inappropriate introduction of too many pseudo-anchor nodes will affect the final estimation result has a serious impact, so it is necessary to optimize the introduced pseudo-anchor nodes.

In this section, we will introduce the fuzzy comprehensive evaluation method to design the location source selection algorithm. For node $j\in N_{i,l}$ to participate in the optimal process of positioning and settlement, the DCPCRLB $posCRLB_{j,l}$, node distance measurement $\;{\tilde{r}}_{ij,l}$, direction angle $\alpha_{ij,l}$ will participate in the judgment as a set of factors:

(10) $$posCRLB_{j,l}=CRLB_{1,1}\left(\theta_{j,l-1}\right)+CRLB_{2,2}\left(\theta_{j,l-1}\right)+Z\cdot c^{2}CRLB_{3,3}\left(\theta_{j,l-1}\right),$$

(11) $$\alpha_{ij,l}=arctan\frac{x_{j,l-1}-x_{i,l-1}}{y_{j,l-1}-y_{i,l-1}},$$

$CRLB_{1,1}\left(\theta_{j,l-1}\right)$ represents the element in the first row and first column of $CRLB\left(\theta_{j,l-1}\right)$, and Z is the scaling constant. The raw data $D_{i,l}$ of the candidate node for node i is:

(12) $$D_{i,l}=\left[\begin{array}{cccc} posCRLB_{1,l} & posCRLB_{2,l} & \cdots & posCRLB_{N,l}\\ {\tilde{r}}_{i1,l} & {\tilde{r}}_{i2,l} & \cdots & {\tilde{r}}_{iN,l}\\ \alpha_{i1,l} & \alpha_{i2,l} & \cdots & \alpha_{iN,l} \end{array}\right],$$

where M is the number of nodes to be selected for node i. Then standardize the data and determine the membership function of each factor according to the number of neighbor anchor nodes and the number of nodes participating in the positioning solution, and perform fuzzy evaluation on the selected nodes to obtain the evaluation matrix $A_{i,l}$:

(13) $$A_{i,l}=\left[\begin{array}{cccc} a_{11} & a_{12} & \cdots & a_{1M}\\ a_{21} & a_{22} & \cdots & a_{2M}\\ a_{31} & a_{32} & \cdots & a_{3M} \end{array}\right],$$

where:

(14) $$a_{1j}=\frac{\underset{j}{\max}posCRLB_{j,l}-posCRLB_{j,l}}{\underset{j}{\max}posCRLB_{j,l}-\underset{j}{\min}posCRLB_{j,l}},$$

(15) $$a_{2j}=\frac{\underset{j}{\max}{\tilde{r}}_{ij,l}-{\tilde{r}}_{ij,l}}{\underset{j}{\max}{\tilde{r}}_{ij,l}-\underset{j}{\min}{\tilde{r}}_{ij,l}},$$

(16) $$a_{3j}=\frac{\underset{j}{\max}\|\varphi_{i,l}-\alpha_{ij,l}\|-\|\varphi_{i,l}-\alpha_{ij,l}\|}{\underset{j}{\max}\|\varphi_{i,l}-\alpha_{ij,l}\|-\underset{j}{\min}\|\varphi_{i,l}-\alpha_{ij,l}\|},$$

Among them, $\varphi_{i,l}$ is jointly determined by the direction angle of neighbor anchor nodes and the number of undetermined sources. The weight function matrix $P_{i,l}=\left[\begin{array}{ccc} w_{1} & w_{2} & w_{3} \end{array}\right]$ can be determined by methods such as entropy weight method [34] and analytical hierarchy process (AHP) [35]. Finally, a fuzzy transformation $Q_{i,l}=P_{i,l}\cdot A_{i,l}$ is performed to obtain the evaluation results of each candidate node, and the one with the largest value is selected as the preferred source for positioning.

**Figure 3:**
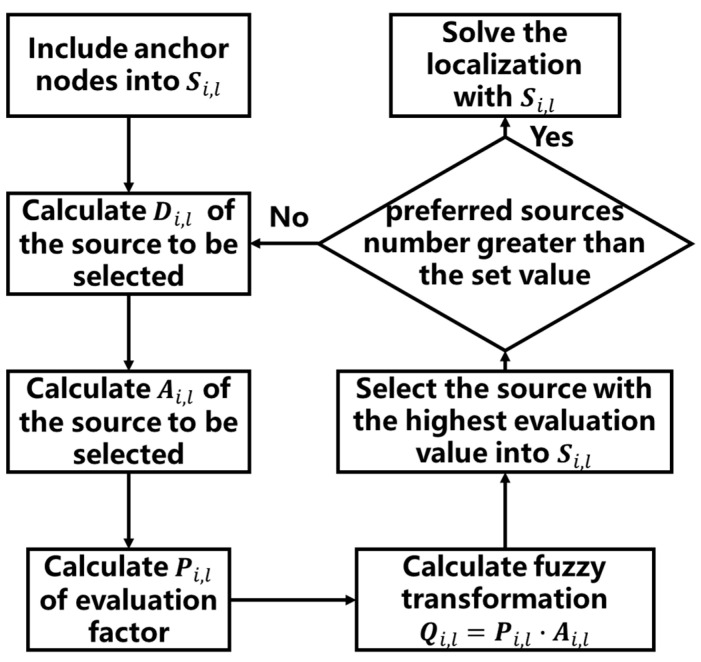
Flow chart of location source selection algorithm based on fuzzy comprehensive evaluation.

As shown in Figure 3, after obtaining neighbor node information and distance measurement, the positioning process enters the positioning source optimization algorithm proposed in this article. First, a certain number of anchor nodes are included in the preferred location source $S_{i,l}$, and then according to Equations (10) and (11) to calculate the raw data $D_{i,l}$ of all other neighbor nodes. The evaluation matrix $A_{i,l}$ is obtained after normalization processing according to Equations (14)–(16), and the weight function matrix $P_{i,l}$ is obtained according to the method mentioned in [34]. Finally, the evaluation value matrix $Q_{i,l}$ of each node is obtained through fuzzy transformation, and the node with the highest evaluation value is selected from it and included in $S_{i,l}$. If the preferred positioning source is sufficient, the location source optimization ends and the positioning solution is entered, otherwise $D_{i,l}$ is recalculated.

## 4. Simulation Scenario and Result Analysis

### 4.1. Simulation Scenario Set

**Figure 4:**
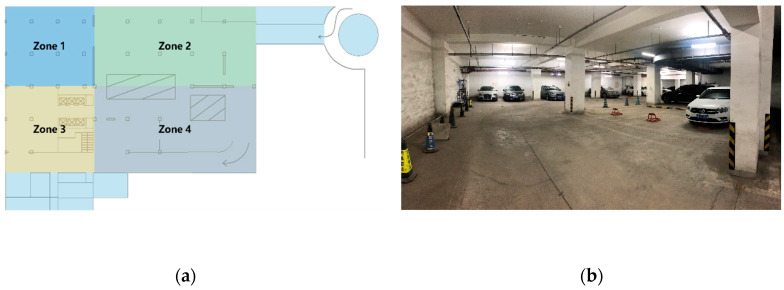
(**a**) Simulation scenario. Zone 1 is the used simulation scene. (**b**) The real scene of zone 1 [22].

In order to make our simulation environment close to the actual scene, the simulation scene will be set according to the zone 1 scene in Figure 4a, which is underground parking lot in Beijing University of Posts and Telecommunications, and Figure 4b is the real scene of zone 1.

The simulation scene is set as a rectangular area of 20 m × 24 m according to the real size of zone 1. To set the average number of anchor node connections around 3, the number of anchor nodes is 4, and the maximum communication distance is set to 10 m. The number of nodes to be located is set to 20 and all nodes conform to the uniform distribution in the simulation scene. All TOA observations are the line of sight (LOS), and the distance measurement error conforms to Gaussian distribution with standard deviation of 0.3 m according to the maximum error of DW1000 (Decawave, Dublin, Ireland). All simulation results are the average of 1000 independent runs.

The initial position measurement error of nodes conforms to the Gaussian distribution, the standard deviation of the anchor node is 0.1 m, the node to be located is 10 m. In the simulation process, the clock slope of node to be located is set as 1 ppm according to the performance of the crystal oscillator used in the hardware (TG5032CFN, EPSON, Nagano-ken, Japan). Anchor nodes are static and the velocity of node to be located is set to 3 m/s as almost the fastest speed of human walking. Root mean square error (RMSE) and cumulative distribution functions (CDF) are used to evaluate the performance of proposed method.

### 4.2. Simulation Result Analysis

**Figure 5:**
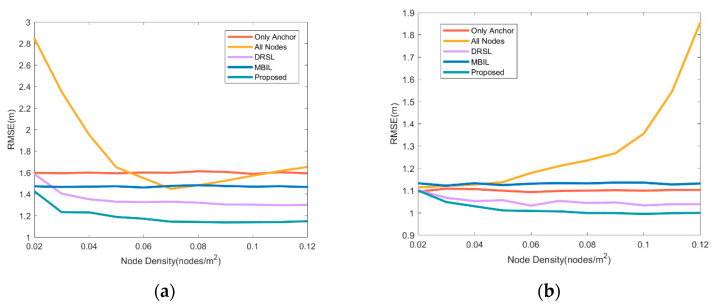
Comparison of positioning accuracy of various source selection methods under different node densities. (**a**) is the comparison when the average number of anchor node connections is 3. (**b**) is the comparison when the average number of anchor node connections is 10. The red line represents the non-cooperative localization using only anchor nodes, the orange represents the cooperative localization using all neighbor nodes as the source, the purple line represents the DRSL source selection location algorithm proposed in [30], the blue line represents the MBIL algorithm in [23] and the green line represents the source optimization algorithm proposed in this article for cooperative localization.

It can be seen from Figure 5 that when the number of neighbor anchor nodes is small, both the DRSL method and the method proposed in this article can effectively improve the positioning accuracy, further increase the number of adjacent anchor nodes, and the positioning source optimization algorithm can significantly improve the positioning accuracy. In addition, the different performance is noticed when all neighbor nodes are used in the graph to participate in positioning. When the neighbor anchor nodes are sufficient, the joining of neighbor cooperative nodes is difficult to improve the positioning accuracy. When the number of neighbor anchor nodes is small, the addition of neighbor cooperative nodes has a positive impact on the positioning accuracy. The proposed algorithm optimizes its role in the positioning process by screening collaborative nodes.

**Figure 6:**
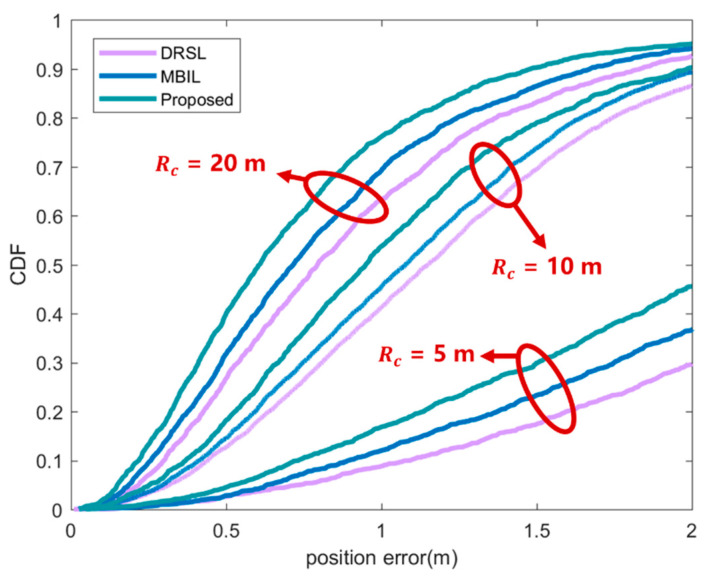
CDF of position error under different communication distance $R_{c}$. In the figure, the purple line represents the DRSL source selection location algorithm proposed in [30], the blue line represents the MBIL algorithm in [23] and the green line represents the source optimization algorithm proposed in this article.

These results in Figure 6 show that the position accuracy affected by communication distance. Obviously, the position accuracy will increase as the communication distance increases. This is because the number of nodes that can be selected increases after the communication distance is increased, and the higher the possibility of selecting a higher-quality node combination, so various methods can obtain better positioning results. At the same time, the position accuracy of the proposed method is better than other methods. This is because the method in this article comprehensively considers many factors that affect the positioning accuracy. The improvement of the communication distance is very effective for improving the positioning accuracy, but as the communication distance increases, the improvement effect of the positioning accuracy becomes smaller. This is because it is difficult for nodes that are too far away to provide effective information for positioning. Therefore, in practical applications, a rigorous analysis of the communication distance, that is, the transmission and reception power, is performed according to the requirements for positioning accuracy, and a reasonable communication distance is obtained.

**Figure 7:**
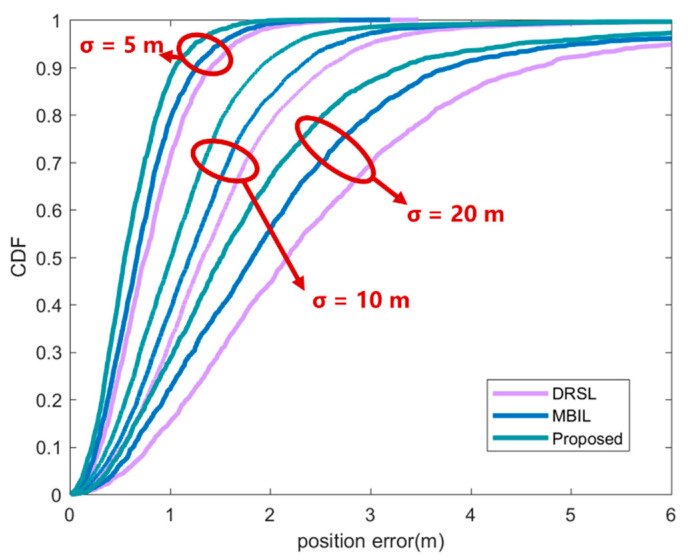
CDF of position error under different initial position error. In the figure, the purple line represents the DRSL source selection location algorithm proposed in [30], the blue line represents the MBIL algorithm in [23] and the green line represents the source optimization algorithm proposed in this article.

Figure 7 shows the positioning accuracy of the node to be located under different initial positioning errors. It can be seen from the figure that the positioning accuracy improves with the decrease of the initial positioning error, because the quality of the position information of the neighboring nodes will affect the positioning accuracy of the nodes. The positioning accuracy of the method proposed in this paper is better than the other two comparison methods. This is because this paper uses DCPCRLB as the evaluation basis for the location information, which has a better estimate of the quality of the location information.

**Figure 8:**
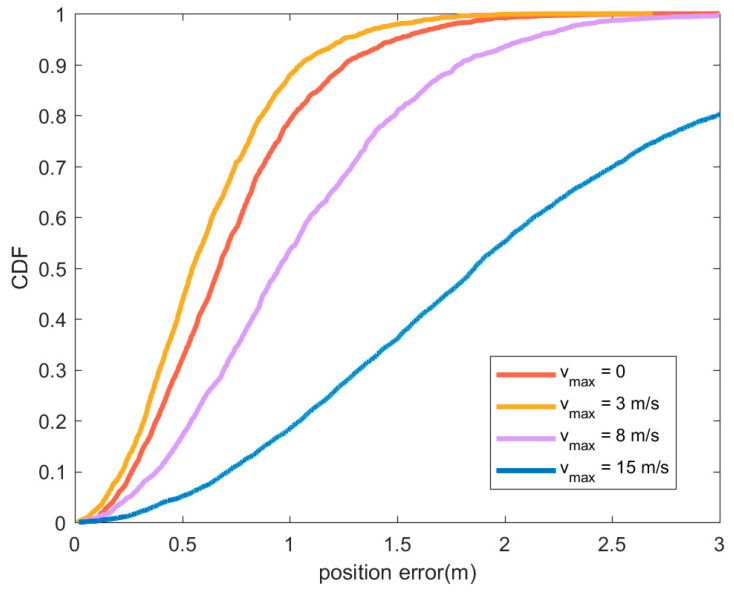
CDF of position error under different maximum node speed $v_{max}$. In the figure, the red line represents the position error CDF when all nodes are static, the orange line represents $v_{max}=3\;m/s$, and the purple line represents $v_{max}=8\;m/s$. The blue line indicates the situation where $v_{max}=15\;m/s$. In all cases, anchor node is static, the nodes to be located conform to the uniform distribution with the maximum speed $v_{max}$, and the direction of movement is random.

In Figure 8, compared with the other three cases, the position accuracy performs best when $v_{max}=3\;m/s$. Although the motion state will cause a certain error in the position estimation of the node to be located, it also has the advantage of changing the topology and optimizing the node distribution. Therefore, when $v_{max}=3\;m/s$, the position accuracy is slightly improved compared to the static situation. When the speed is too high ($v_{max}=8\;m/s$ or $v_{max}=15\;m/s$), the error caused by the movement cannot be compensated by optimizing the node distribution. The error caused by motion can be compensated by combining positioning with sensor data such as accelerometer or gyroscope [36].

**Figure 9:**
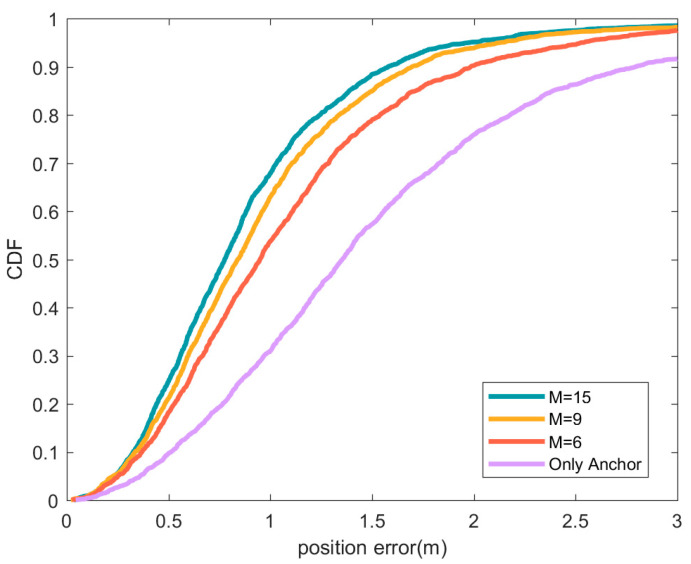
CDF of position error under different number of preferred positioning sources. In the figure, the green line represents the location error CDF when the preferred location source is 15, the orange line represents the preferred location source is 9, and the red line represents the preferred location source is 6. The purple line indicates the situation where only anchor nodes are used for positioning.

Figure 9 compares the positioning performance when using different numbers of preferred position sources in a low anchor node density scenario. It can be seen that as the number of preferred positioning sources increases, the position accuracy is improved. As the number of preferred positioning sources increases, the signal quality of the position sources and the geometric distribution of the position sources will be improved, and the position accuracy will be improved to a certain extent, but the improvement effect will decrease as the number of equipotential sources increases. By comparing the results with Figure 5, it can be found that there is a limit to improving the positioning effect by increasing the number of position sources. Too many position sources may also cause a decrease in position accuracy, which is the fact reflected by the yellow line in Figure 5.

**Figure 10:**
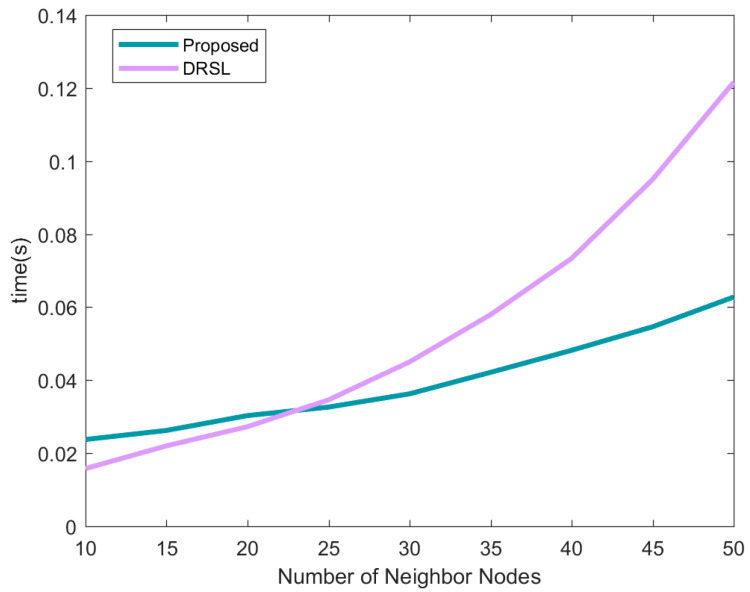
The average positioning time of each node under different numbers of neighbor nodes.

Figure 10 reflects the average positioning time of the proposed method and DRSL method under different numbers of neighbor nodes. The time complexity of the location source optimization algorithm is mainly related to both the average number of neighbor nodes $N_{1}$ and the number of preferred sources $N_{2}$. For the calculation of the original data $D_{i,l}$, the evaluation matrix $A_{i,l}$ and the fuzzy transformation $Q_{i,l}$ in the algorithm proposed in this paper, the time complexity is $2O\left(N_{1}\right)$, $5O\left(N_{1}\right)$ and $3O\left(N_{1}\right)$. Finally, a preferred positioning source is obtained after comparison. Repeating the above process $N_{2}$ times is the time complexity of the node. By optimizing part of the algorithm process, the time complexity is finally $5O\left(N_{1}N_{2}\right)$. In the proposed method, nodes transmit a localization vector including $\theta_{i,l}={\left[\begin{array}{ccc} x_{i,l} & y_{i,l} & a_{i,l} \end{array}\right]}^{T}$ and a the DCPCRLB $posCRLB_{i,l}$, so the communication cost is 2O(1). The computational complexity, run-time and communication overhead of the three algorithms is shown in Table 2.

### 4.3. Future Research Directions

In the next stage of research, the following aspects will be mainly focused on: First, we plan to analyze other factors which affect position accuracy. Secondly, we shall implement the proposed method based on hardware platform and apply the proposed method in realistic scenarios. The measured results in realistic scenarios will be compared with the simulation results to improve the performances of the proposed method.

## 5. Conclusions

We propose a new positioning source optimization method for low-anchor node density wireless sensor networks, which comprehensively considers the positioning performance, distance, location and other factors that affect the positioning accuracy of cooperative nodes to select positioning sources. First, each node calculates its own CRLB and transmits it to neighboring nodes through collaborative information. Neighboring nodes calculate the CRLB, distance measurement and direction angle of neighboring cooperative nodes to obtain the optimal evaluation matrix, and obtain the fuzzy evaluation result through weight addition, and obtain the final optimal location source. In the fuzzy comprehensive evaluation framework, the weights of various evaluation factors can be flexibly configured. Therefore, the proposed method can screen out different neighbor nodes as positioning sources according to requirements, and then obtain a combination of positioning sources with good location performance and distribution, which can improve the positioning accuracy of the node. Compared with the DRSL and MBIL method, the positioning signal source obtained by this method can obtain higher positioning accuracy about 33.9% and 19.4% under low anchor node density, and the sacrificed calculation time is almost negligible.

## Figures and Tables

**Table 1:** Comparative of different location source selection algorithms.

Location Source Selection Algorithm in Localization	Factors of Location Source Selection	Method of Fusion between Factors
MBIL [23]	the number of iterations, the rediual energy and the deviation degree error	linear combination
LSL-DC [24]	the distance measure error	single factor
NS-IPSO [25]	the distance measure error and the communication frequency	linear combination
BASL [26]	the number of boundary region nodes	single factor
GSTDOA [27]	HDOP	single factor
enhanced three-dimensional DV-hop [28]	coplanarity	single factor
SNA-CC [29]	closeness centrality	single factor
DRSL [30]	the smallest least square error	single factor
Proposed method	the DCPCRLB, the distance measurement and direction angle	the fuzzy comprehensive evaluation

**Table 2:** Comparisons of different methods for each node at each iteration.

Method	Computational Complexity	Run-Time	Communication Overhead
Proposed method	$5O\left(N_{1}N_{2}\right)$	42.319 ms	2×O(1)
DRSL [30]	$O\left(N_{1}^{2}\right)+2O\left(N_{2}\right)$	84.532 ms	$O\left(N_{2}\right)$+ O(1)
MBIL [23]	$O\left(N_{1}N_{2}^{2}\right)$	326.443 ms	$O\left(N_{1}\right)+2\times O\left(N_{1}\right)$

## Data Availability

Not applicable.

## References

[1] Capella J.V., Campelo J.C., Bonastre A., Ors R. (2016). A reference model for monitoring IoT WSN-based applications. Sensors.

[2] Carvalho C., Gomes D.G., Agoulmine N., De Souza J.N. (2011). Improving prediction accuracy for WSN data reduction by applying multivariate spatio-temporal correlation. Sensors.

[3] Lu W., Gong Y., Liu X., Wu J., Peng H. (2017). Collaborative energy and information transfer in green wireless sensor networks for smart cities. IEEE Trans. Ind. Inf..

[4] Bagula A.B., Osunmakinde I., Zennaro M. (2010). On the relevance of using Bayesian belief networks in wireless sensor networks situation recognition. Sensors.

[5] Kumar S.A., Ilango P. (2018). The impact of wireless sensor network in the field of precision agriculture: A review. Wirel. Wirel. Pers. Commun..

[6] Adame T., Bel A., Carreras A., Melia-Segui J., Oliver M., Pous R. (2018). CUIDATS: An RFID–WSN hybrid monitoring system for smart health care environments. Future Gener. Comput. Syst..

[7] Boubrima A., Bechkit W., Rivano H. (2017). Optimal WSN deployment models for air pollution monitoring. IEEE Trans. Wirel. Commun..

[8] Han G., Yang X., Liu L., Zhang W., Guizani M. (2017). A disaster management-oriented path planning for mobile anchor node-based localization in wireless sensor networks. IEEE Trans. Emerg. Top. Comput..

[9] Radmand P., Talevski A., Petersen S., Carlsen S. Comparison of industrial WSN standards. Proceedings of the 4th IEEE International Conference on Digital Ecosystems and Technologies.

[10] Kenniche H., Ravelomananana V. Random geometric graphs as model of wireless sensor networks. Proceedings of the 2010 2nd International Conference on Computer and Automation Engineering (ICCAE).

[11] Elhoseny M., Hassanien A.E. (2019). Extending homogeneous WSN lifetime in dynamic environments using the clustering model. Dynamic Wireless Sensor Networks.

[12] Popescu D., Dragana C., Stoican F., Ichim L., Stamatescu G. (2018). A collaborative UAV-WSN network for monitoring large areas. Sensors.

[13] Elhoseny M., Hassanien A.E. (2019). Expand mobile WSN coverage in harsh environments. Dynamic Wireless Sensor Networks.

[14] Yuan Y., Huo L., Wang Z., Hogrefe D. (2018). Secure APIT localization scheme against sybil attacks in distributed wireless sensor networks. IEEE Access.

[15] Cheikhrouhou O., M Bhatti G., Alroobaea R. (2018). A hybrid DV-hop algorithm using RSSI for localization in large-scale wireless sensor networks. Sensors.

[16] Yoo J. (2019). Change Detection of RSSI Fingerprint Pattern for Indoor Positioning System. IEEE Sens. J..

[17] Li G., Geng E., Ye Z., Xu Y., Lin J., Pang Y. (2018). Indoor positioning algorithm based on the improved RSSI distance model. Sensors.

[18] Wu S., Zhang S., Huang D. (2019). A TOA-based localization algorithm with simultaneous NLOS mitigation and synchronization error elimination. IEEE Sens. Lett..

[19] Díez-González J., Álvarez R., Sánchez-González L., Fernández-Robles L., Pérez H., Castejón-Limas M. (2019). 3D Tdoa problem solution with four receiving nodes. Sensors.

[20] Zhang L., Chen M., Wang X., Wang Z. (2018). TOA estimation of chirp signal in dense multipath environment for low-cost acoustic ranging. IEEE Trans. Instrum. Meas..

[21] Wymeersch H., Lien J., Win M.Z. (2009). Cooperative localization in wireless networks. Proc. IEEE.

[22] Deng Z., Tang S., Jia B., Wang H., Deng X., Zheng X. (2020). Cooperative Localization and Time Synchronization Based on M-VMP Method. Sensors.

[23] Su Y., Guo L., Jin Z., Fu X. (2020). A Mobile-beacon Based Iterative Localization Mechanism in Large-scale Underwater Acoustic Sensor Networks. IEEE Internet Things J..

[24] Chen X., Chen J., Chen C., He J., Lei B. (2013). A node localization algorithm for wireless sensor networks using distance clustering to select the anchor nodes. Sens. Lett..

[25] Phoemphon S., So-In C., Leelathakul N. (2020). A hybrid localization model using node segmentation and improved particle swarm optimization with obstacle-awareness for wireless sensor networks. Expert Syst. Appl..

[26] Zhang K., Zhang G., Yu X., Hu S. (2021). Boundary-Based Anchor Selection Method for WSNs Node Localization. Arab. J. Sci. Eng..

[27] Sun S., Qin S., Hao Y., Zhang G., Zhao C. (2020). Underwater Acoustic Localization of the Black Box Based on Generalized Second-Order Time Difference of Arrival (GSTDOA). IEEE Trans. Geosci. Remote Sens..

[28] Kaushik A., Lobiyal D.K. (2019). Enhanced Three-Dimensional DV-Hop Algorithm. ICT Systems and Sustainability.

[29] Ahmad T., Li X.J., Seet B.-C., Cano J.-C. (2020). Social network analysis based localization technique with clustered closeness centrality for 3d wireless sensor networks. Electronics.

[30] Gao J., Shen X., Mei H., Zhang Z. (2019). Dynamic Reference Selection-Based Self-Localization Algorithm for Drifted Underwater Acoustic Networks. Sensors.

[31] Zhang T.N., Li J.L. (2002). Application of multi-step fuzzy comprehensive evaluation. J. Harbin Eng. Univ..

[32] Barker A.L., Brown D.E., Martin W.N. (1995). Bayesian estimation and the Kalman filter. Comput. Math. Appl..

[33] Zheng Y., Ozdemir O., Niu R., Varshney P.K. (2012). New Conditional Posterior Cramér-Rao Lower Bounds for Nonlinear Sequential Bayesian Estimation. IEEE Trans. Signal Process..

[34] Anees J., Zhang H.-C., Baig S., Guene Lougou B., Robert Bona T.G. (2020). Hesitant Fuzzy Entropy-Based Opportunistic Clustering and Data Fusion Algorithm for Heterogeneous Wireless Sensor Networks. Sensors.

[35] Wang Y.-M., Luo Y., Hua Z. (2008). On the extent analysis method for fuzzy AHP and its applications. Eur. J. Oper. Res..

[36] Luo J., Zhang C., Wang C. (2020). Indoor multi-floor 3D target tracking based on the multi-sensor fusion. IEEE Access.

